# Collagen’s Triple Helix Reduces the Susceptibility to Base-Catalyzed Hydrolysis

**DOI:** 10.1021/acsomega.6c05284

**Published:** 2026-08-10

**Authors:** Jannik Buhr, Frauke Gräter

**Affiliations:** † 28308 Heidelberg Institute for Theoretical Studies, Am Schloss-Wolfsbrunnenweg 35, Heidelberg 69117, Germany; ‡ Institute for Scientific Computing, 40092 Heidelberg University, Im Neuenheimer Feld 205, Heidelberg 69120, Germany; § Max Planck Institute for Polymer Research, Ackermannweg 10, Mainz 55128, Germany; ∥ Max Planck School Matter to Life, Jahnstraße 29, Heidelberg 69120, Germany

## Abstract

Collagen is the most abundant structural protein in mammals. As the primary component of tendons, ligaments, and other connective tissues, it has to withstand extreme forces. Base-catalyzed peptide hydrolysis was recently shown to be greatly accelerated by mechanical force. This puts collagen at risk of degradation under physiological loads, yet collagen is known to be remarkably stable. Using hybrid quantum mechanics/molecular mechanics (QM/MM) simulations, we investigated the hydrolysis reaction within the complex chemical environment of a collagen triple helix. We demonstrate that the unique triple-helical structure increases the reaction barrier for base-catalyzed hydrolysis compared to a single peptide chain. Our results can explain why collagen mechanical failure proceeds through homolytic bond rupture and mechanoradical formation.

## Graphical abstract

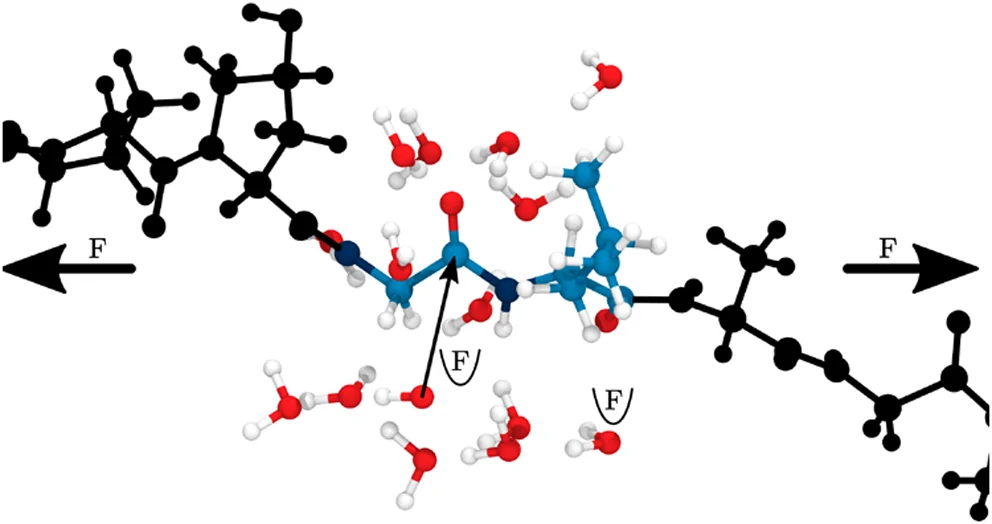

## Introduction

Collagen is the predominant protein in mammals,^1^ constituting the bulk of connective tissue, cartilage, skin, and tendons. Its mechanical role derives from a striking hierarchical organization that spans several orders of magnitude in length scale.^2,3^ Three polypeptide chains, each built from characteristic Gly-X-Y repeats, wind into a right-handed triple helix. These molecules associate laterally and stagger axially into microfibrils and fibrils with the characteristic D-periodic banding (Figure 1a, Orgel et al., 2006), which in turn bundle into fibers and fascicles. The high abundance of glycine, proline, and hydroxyproline is essential to this packing. Glycine, the smallest amino acid, occupies the crowded helix core, while the pyrrolidine rings of proline and hydroxyproline lock the backbone Φ dihedral near −65°, conferring rigidity. The stereoelectronic consequences of 4R-hydroxylation further stabilize the fold.^3–5^ Together, this architecture renders collagen an exceptionally stable structural protein.

**Figure 1:**
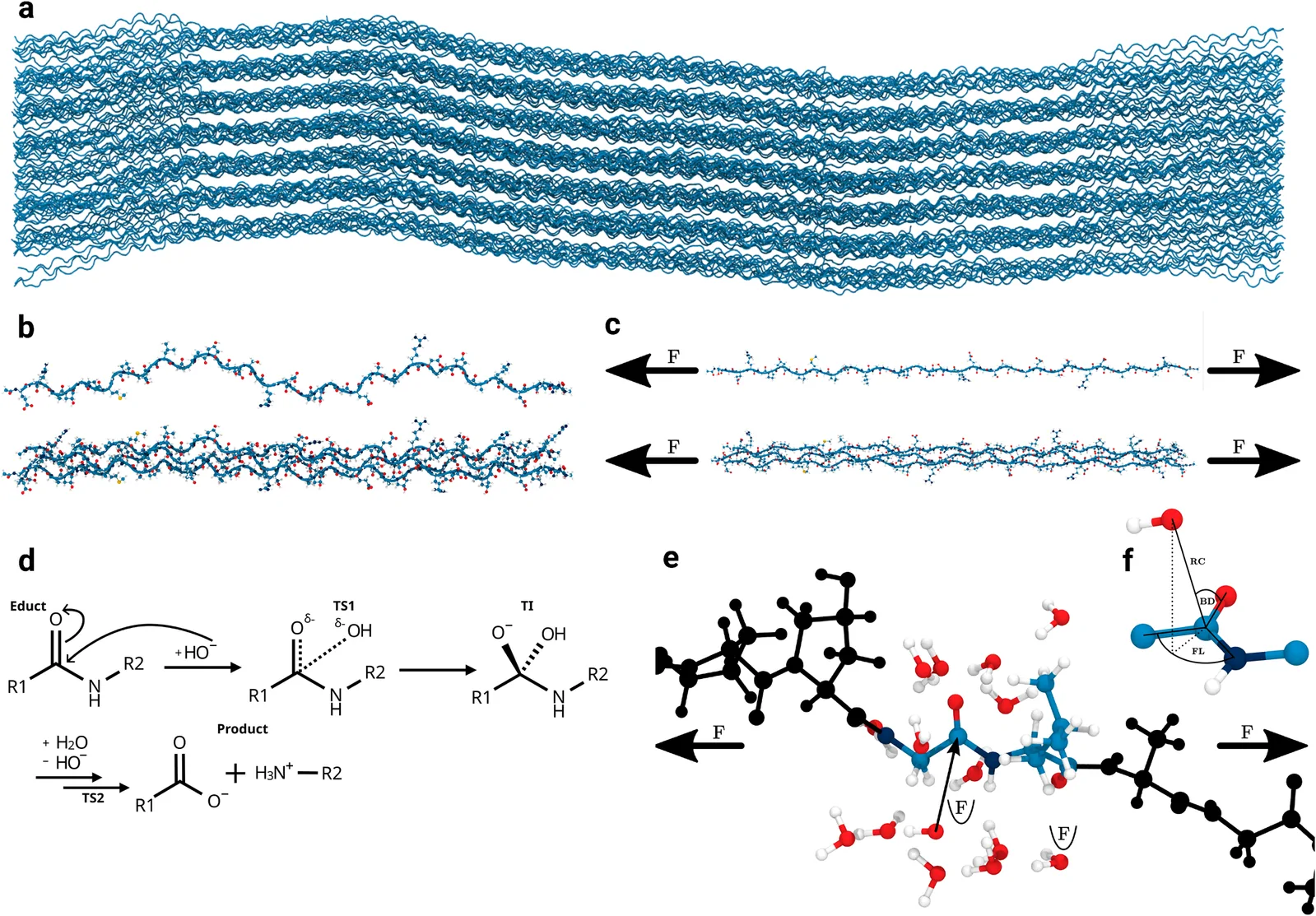
Simulation setup and reaction scheme for base-catalyzed peptide bond hydrolysis. (a) Structure of a collagen type I fibril. (b) A single peptide (chain C) and the triple helix before equilibration under force. (c) A single peptide (chain C) and the triple helix after equilibration under 1 nN of external force. (d) Reaction scheme of base-catalyzed peptide bond hydrolysis. TS: transition state; TI: tetrahedral intermediate; ZI: zwitterionic state. (e) The QM subsystem (color) embedded in the MM system (black) with force vectors shown at the start of the hydroxide attack (Gly-Leu site). MM water molecules are omitted for clarity. (f) Scheme of the Bürgi-Dunitz (BD) and Flippin-Lodge (FL) angles characterizing the nucleophilic attack. The reaction coordinate of transition state 1 (TS1) is defined as the distance between the carbonyl carbon and the attacking hydroxide oxygen and labeled as RC.

In tissues such as tendons, collagen is subjected to significant mechanical stress.^6^ For example, hamstring tendons can transfer forces up to 1 kN,^7^ resulting in macroscopic stresses of 10 MPa. Considering that the effective load-bearing cross-sectional area (excluding water content of ∼60%^8^) is much smaller, the molecular forces are considerably higher and in principle can reach Nanonewtons as required for covalent bond opening by force. A large body of experimental and computational work has dissected how collagen accommodates such loads across its hierarchy through molecular stretching and uncoiling, interfibrillar sliding, and the gradual loss of interactions.^9–12^ Atomistic simulations in particular have shown that, at the molecular scale, tensile loads localize onto individual backbone bonds and can reach the regime where covalent chemistry is activated.^10,11,13^ Understanding how such molecular forces influence the chemical stability of the collagen backbone is therefore central to explaining durability and failure of connective tissue.

How mechanical force impacts chemical reactions has been described by Bell and Eyring-type kinetic models,^14,15^ and quantified by single molecule force spectroscopy.^16^ The two major reaction pathways by which a protein like collagen can undergo covalent bond scission are homolytic bond rupture, leading to radicals, and heterolytic bond rupture, i.e., hydrolysis of the peptide bond. Which of these pathways dominate under mechanical force governs the chemical products and downstream biological consequences, but is not fully understood. On one hand, mechanical strain modulates cleavage by matrix metalloproteinases and bacterial collagenases, which catalyze hydrolysis. Tension generally shields fibrillar collagen from proteolysis, whereas under other loading geometries force can accelerate cleavage of individual molecules.^17–20^ A previous study showed that peptide hydrolysis is strongly accelerated by mechanical force, using atomic force microscopy and quantum mechanical (QM) calculations.^21^ On the other hand, we recently could show that mechanoradicals stemming from homolysis can be observed in stretched collagen.^22^ Interestingly, a previous experimental study of small proline esters as model compounds suggests that collagen is less prone to base-catalyzed peptide hydrolysis due to a *n* → π* stabilizing interaction between adjacent carbonyl groups, an interaction which is prevalent in the triple helix secondary structure.^23^ Taken together, both pathways can in principle occur in stretched collagen and compete with each other, thereby determining biochemical outcomes.

Base-catalyzed hydrolysis breaks a peptide bond via nucleophilic attack by a hydroxide ion (OH^–^) (Figure 1d,e). The mechanism proceeds through several steps: (1) The catalytic hydroxide attacks the electrophilic carbonyl carbon, overcoming the first energy barrier (TS1) to form a tetrahedral intermediate (TI). The TI is stabilized by hydrogen bonds with solvent molecules or the protein backbone. (2) Rupture of the peptide bond (TS2) results in separate C- and N-termini. Overall, one water molecule is added to the peptide during hydrolysis. The transition states and intermediates are well characterized by quantum chemical studies of amide and peptide hydrolysis in solution.^24^ In parallel, a range of computational mechanochemistry methods now make it possible to model bond rupture under load from first-principles, including the constrained-geometries-simulate-external-force (CoGEF) approach^25^ or related schemes.^26,27^ Crucially, however, almost all previous treatments of force-accelerated peptide hydrolysis have relied on minimized single-point structures of isolated peptides or small model compounds, neglecting the fluctuating, densely hydrogen-bonded, and sterically crowded environment that the collagen triple helix actually presents to an incoming nucleophile. Whether and how strongly this native environment modulates the hydrolysis barrier has remained unresolved.

The goal of this study is to explore hydrolysis within the complex, dynamical environment of triple helices found in collagen. Our approach of using large-scale QM/MM simulations where the QM region encompasses two complete residues and surrounding water molecules complements the single-point energies of optimized structures reported by Pill et al.^21^ The reaction is directly sampled in a realistic, fluctuating environment, providing insight into the reaction dynamics. We hypothesize that the triple helical structure of collagen hinders the approach of the hydroxide ion, thereby energetically disfavoring the formation of the tetrahedral intermediate. Our results have implications for understanding the chemistry that underlies collagen mechanical degradation.

## Methods

Simulations and analyses were managed by a custom automated pipeline available at github.com/graeter-group/hydrolysis-qmmm-workflow. The workflow, implemented in Python, controlled interactions with the molecular dynamics engine GROMACS^28–30^ (v2023.2) and the CP2K quantum chemistry package^31^ (v2023.2). Although further steps of the hydrolysis reaction are also covered by the workflow, we here focus on the nucleophilic attack of the hydroxide ion and its barrier. This step has been shown to be the rate-limiting step of hydrolysis under a stretching force as considered here.^21^ In the following, the relevant steps are described in methodological detail, while the code is provided in the repository linked above for exact reproducibility.

### Initial Structure Preparation

The initial triple helix structure (Figure 1a) was excised from a *Homo sapiens* collagen type I fibril (without cross-links) from ColBuilder.^32^ It comprises three chains (A, B, and C) of 45, 43, and 45 amino acids, respectively. The N-termini were acetyl-capped (ACE) and C-termini were *N*-methyl capped (NME) using PyMOL.^33^

### Equilibration Protocol

Structures were converted to GROMACS topologies using the amber99sb*-ildnp force field.^34–36^ Partial charges for the hydroxide ion were adapted from CHARMM36.^37^ The protein was placed in a box with a minimum 2 nm buffer, scaled by 130% to accommodate pulling, solvated in TIP3P water, and neutralized with 0.15 M NaCl. Following energy minimization with the steepest descent algorithm, the system was equilibrated subsequently in the NVT and NPT ensemble with position restraints on the protein for 100 ps each. Temperature coupling was applied using the velocity rescaling thermostat^38^ at 310 K. Pressure coupling was applied isotropically using the Parrinello–Rahman barostat.^39^

Each system was further equilibrated under an external force of 1 nN per chain (603 kJ/mol/nm), which was applied to the center of mass (COM) of the capping residues’ α-carbons in opposing directions, for 100 ps in the NPT ensemble. The triple helix included rotational restraints to prevent unwinding, mimicking the environment within a fibril. The equilibration duration was validated by monitoring the pull group distances (Figure 2f).

**Figure 2:**
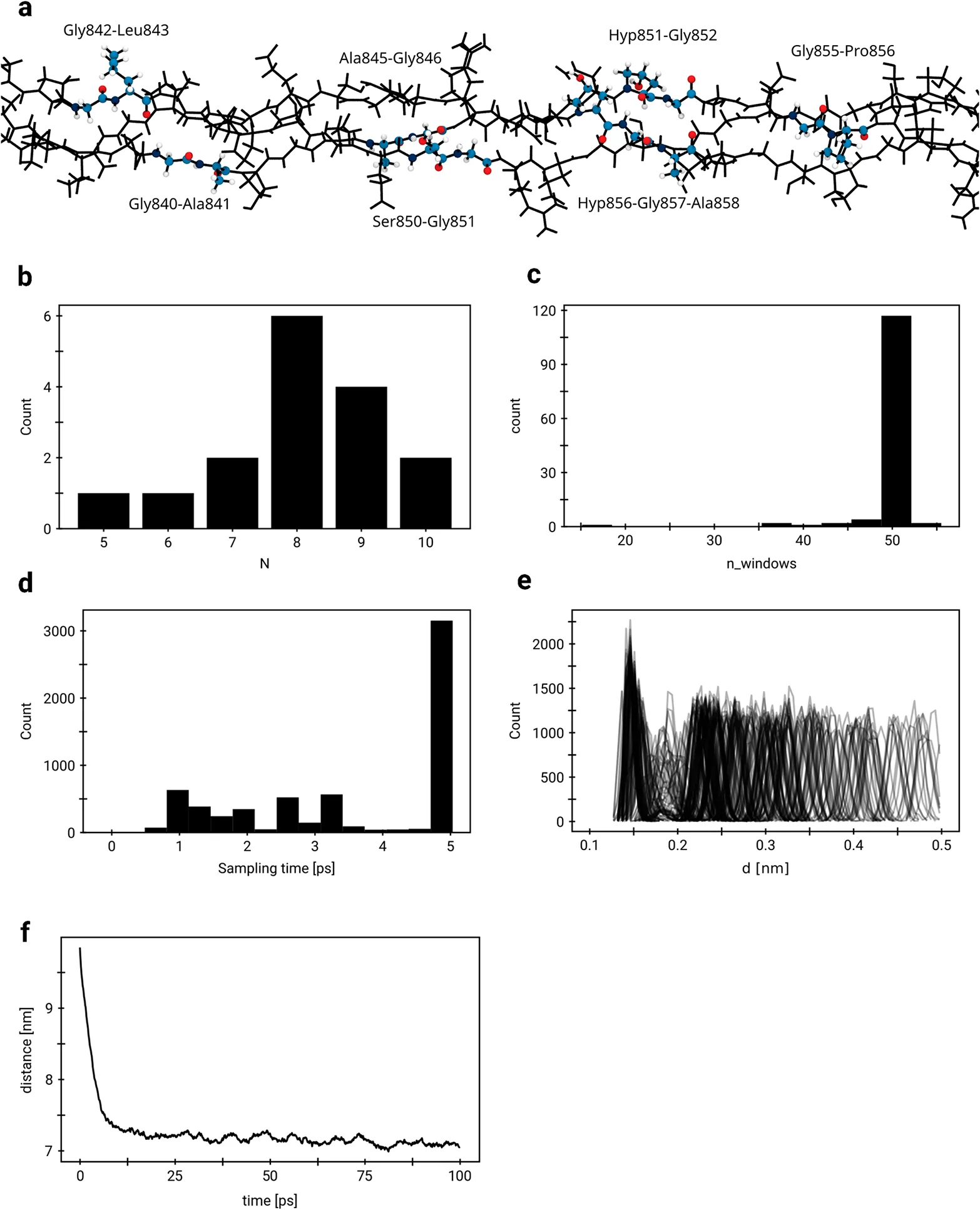
Details on the simulations and umbrella sampling of the nucleophilic attack leading to TI. (a) Close-up of the sampled sites in triple helix and single chain configurations. QM regions (residues involved in the peptide bond) are shown in ball-and-stick; the protein backbone is shown as lines. (b) Histogram of successful hydroxide attack simulations (out of 10 starts). (c) Histogram of umbrella sampling windows generated per trajectory. Rarely, instead of the TI formation, a substitution reaction occurred wherein the attacking hydroxide displaced the carbonyl oxygen. This rare event necessitated filtering; 62 out of 6359 umbrella windows were discarded. Windows were excluded if the average carbonyl C–O distance exceeded 0.155 nm (substitution) or if the sum of C–O (carbonyl) and C–O (hydroxide) distances was less than 0.277 nm (collapsing TI). (d) Histogram of umbrella window simulation durations. All windows combined total a sampling time of 22.7 ns. (e) Coverage of umbrella windows along the TS1 reaction coordinate. (f) Pull group distance vs time during triple helix equilibration (1 nN).

### Quantum Mechanics Subsystem Selection

The last 10 frames of the force equilibration were extracted to provide starting configurations for the production simulations. Eight peptide bonds near the center of the triple helix were selected to avoid end effects (Figure 2a). For each site, the workflow identified candidate water molecules for the initial attack (TS1, see Figure 1d).

The optimal attack trajectory was determined using the Bürgi-Dunitz (BD) angle (107°)^40^ and Flippin-Lodge (FL) angle.^41^ A penalty function (eq 1) was applied to the 15 solvent molecules closest to the carbonyl carbon

1 $$p=\sqrt{{\left(\mathrm{BD}-107\right)}^{2}+{\left(\mathrm{FL}-0\right)}^{2}}$$

The water molecule with the lowest penalty was transformed into the nucleophilic hydroxide by removing one proton. Neutrality of the system was maintained by removing a Cl^–^ ion from the MM subsystem. The hydroxide, along with the remaining 14 closest solvent molecules and the atoms of the two target residues, formed the QM region. To prevent exchange with MM water, a harmonic potential kept the QM water COM centered on the peptide bond. The effectiveness of this potential was verified by monitoring distances of the reaction site to the MM and QM solvent molecules (Figure 3).

**Figure 3:**
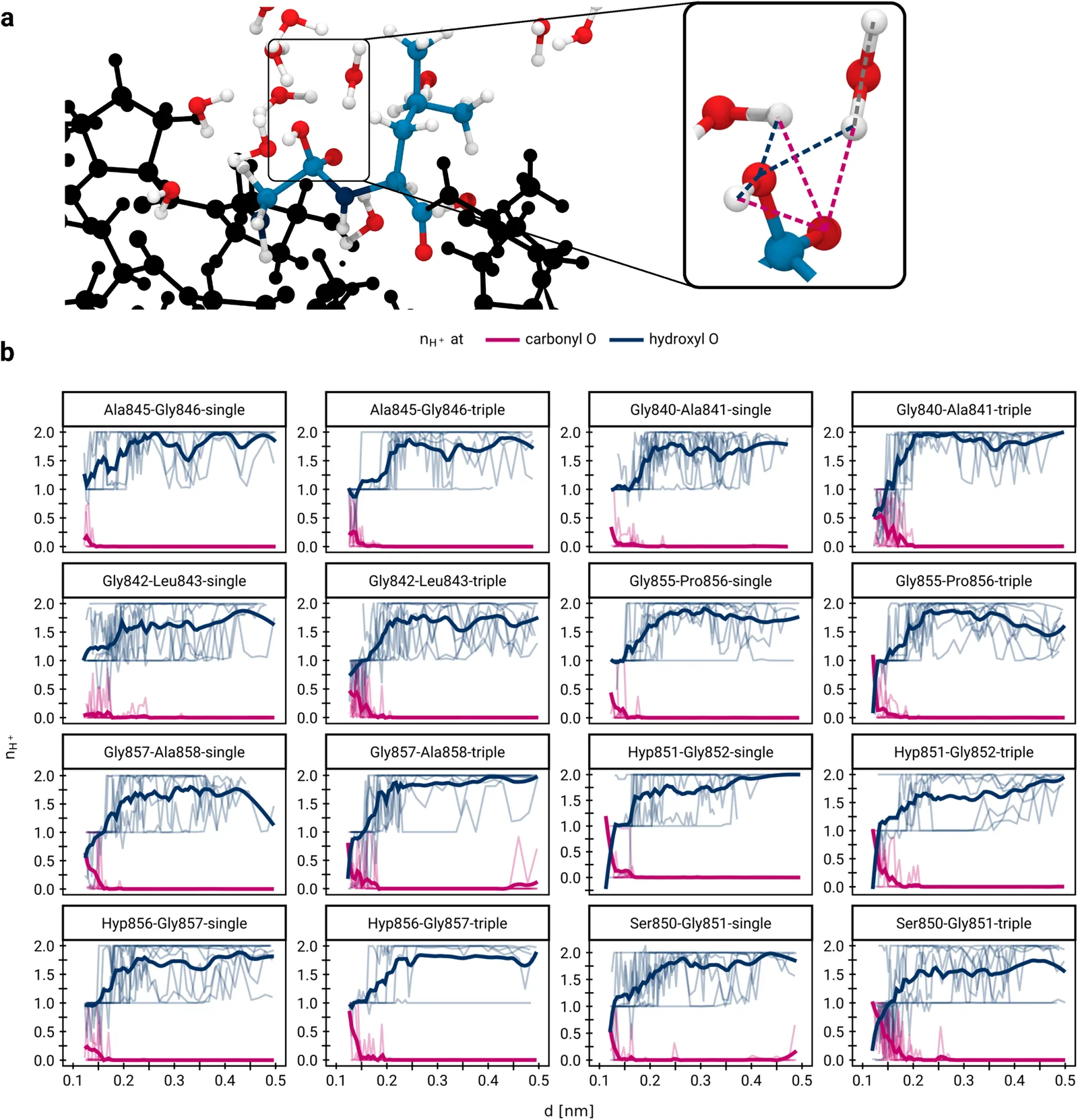
Mechanistic details of the tetrahedral intermediate formation. (a) The tetrahedral intermediate (TI) state stably formed in a QM/MM simulation of the hydroxide attack in the triple helix system at a glycine-leucine site. The QM subsystem is shown in color embedded in the MM system (black). MM water molecules are omitted. (b) Protonation states of the carbonyl O of the TI and approaching hydroxide during formation. Protons were uniquely assigned to the nearest proton acceptor. Data points represent averages within each window; thick lines indicate LOESS curves.

A 100 step QM/MM warm-up (Δ*t* = 0.2 fs) with an umbrella potential on the hydroxide stabilized the system before the approach trajectory. QM calculations utilized the TZV2P-MOLOPT-GTH basis set^42^ and the PBE functional,^43,44^ which was found to adequately describe the charge delocalization and proton transfer reactions. We used a variant of the electrostatic embedding during the QM/MM simulations, i.e., the default setting of the CP2K-Gromacs interface (called GEEP^45^). QM/MM simulations were run in the NVT ensemble.

### Umbrella Sampling

Umbrella sampling was performed to calculate free energies along the reaction coordinate the distance between the attacking hydroxide oxygen and the carbonyl carbon of the peptide bond (Figure 1e,f).

To this end, the approach was simulated with a 1 fs time step (approximately 2 ps duration) using a harmonic force constant of 40,000 kJ/mol/nm^2^. The constant force applied to the simulation systems during the equilibration (see above) was maintained during the umbrella simulations. For each reaction site, up to ten (Figure 2b) attack trajectories were simulated, each starting from a different frame of the force-equilibrated system. From each initial trajecotyry, 50 snapshots were extracted: 30 equidistant below 0.25 nm (to sample the transition state) and 20 above. The sampling runs started from these snapshots used a force constant of 50,000 kJ/mol/nm^2^ and where simulated for up to 5 ps (Figure 2d). For analysis, sampling windows for each site were pooled from multiple initial trajectories to enhance phase space coverage. Data were analyzed using the Weighted Histogram Analysis Method (WHAM)^46,47^ starting at 0.05 ps (50 steps) using 80 bins and 5 bootstraps. Additional statistics on the umbrella sampling runs are shown in Figure 2b–e.

## Results

### Mechanistic Details of Tetrahedral Intermediate Formation

We selected 8 hydrolysis sites along a triple helix such that different pairs of amino acids adjacent to the cleaving peptide bond are sampled. All selected hydrolysis sites (Figure 2a) yielded at least 5 successful hydroxide attack trajectories (defined as reaching a C–O distance <0.145 nm without crashing), with some sites achieving 100% success (Figure 2b). From most trajectories, we could successfully initiate the 50 umbrella sampling windows (Figure 2c), and the majority of sampling simulations completed the full 5 ps duration per window (Figure 2d).

Analysis of proton assignments reveals the dynamic nature of the reaction (Figure 3). As the hydroxide subjected to the umbrella potential approaches the carbonyl group, the system fluctuates between the hydroxide ion, as originally placed in the simulation system, and a water molecule due to proton exchange with the other QM solvent molecules. While the initial hydroxide is far away from the peptide bond, it is equally likely to be protonated as other solvent molecules, i.e., the oxygen carries close to two hydrogens on average. Frequent oscillations between one and two hydrogens are observed for all simulated hydrolysis sites, reflecting sufficient sampling to cover frequent protonation state changes across the QM water, as expected.

**Figure 4:**
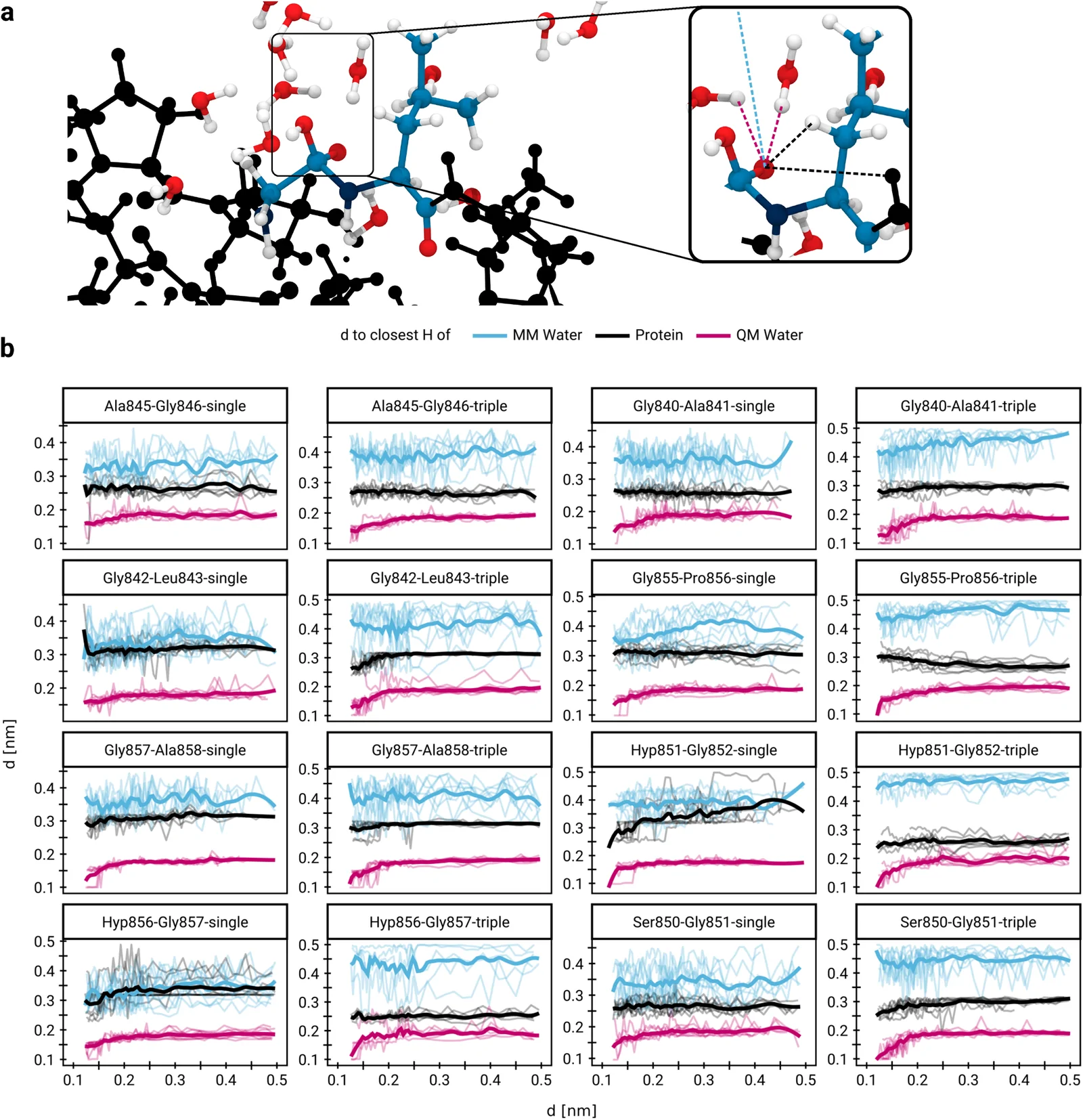
Stabilization of the tetrahedral intermediate by surrounding proton donors. (a) TI state in the triple helix (Gly-Leu site). QM subsystem in color; MM system in black. (b) Distance from the TI carbonyl oxygen to the nearest proton of: MM solvent (cyan), protein (black), or QM water (magenta). Data are averaged across windows; thick lines represent LOESS curves.

As the distance to the carbonyl carbon during nucleophilic attack decreases below 0.2 nm, the attacking molecule is more likely to be deprotonated, i.e., favors the hydroxide form over water during attack. This handoff of the proton can happen at various points on the reaction coordinate, i.e., for various distances of the attacking oxygen from the carbonyl carbon, indicated by the individual trajectory lines, stressing the dynamic nature of the reaction. The availability of a proton acceptor near the attacking hydroxide is crucial for overcoming the energy barrier to form the TI. Also, upon formation of the tetrahedral intermediate (TI, Figures 1 and[Fig fig2]a), the increased negative charge density at the carbonyl oxygen is stabilized by a hydrogen bond network with the surrounding water (Figure 4). Consistently across the hydrolysis sites and for both single peptides and triple helix simulations, the carbonyl oxygen is hydrogen bonded to water protons. Hydrogen bond donors of the protein instead are not directly involved in the stabilization (distances of 0.25 nm or larger), at least across the set of hydrolysis sites selected here.

As shown in Figure 5, the N atom of the peptide bond is also taking part in the overall proton mobility as a proton acceptor in the sense that is is occasionally sharing or exchanging a proton with a solvent molecule, but no spontaneous formation of a zwitterionic state (i.e., a positive charge on the N) can be observed during sampling.

Eventually, the carbonyl oxygen, with its stronger negative charge density in the TI, attracts a proton (Figure 3). On average across all trajectories, this protonation occurs in parallel with the deprotonation of the attacking water, i.e., for *d* below 0.2 nm, resulting in an average of one proton on the two oxygen atoms of the TI. These proton transfer steps do not always occur prior or after the C–O bond formation, but rather in parallel, i.e., are coupled to the nucleophilic attack, according to our simulation results.

**Figure 5:**
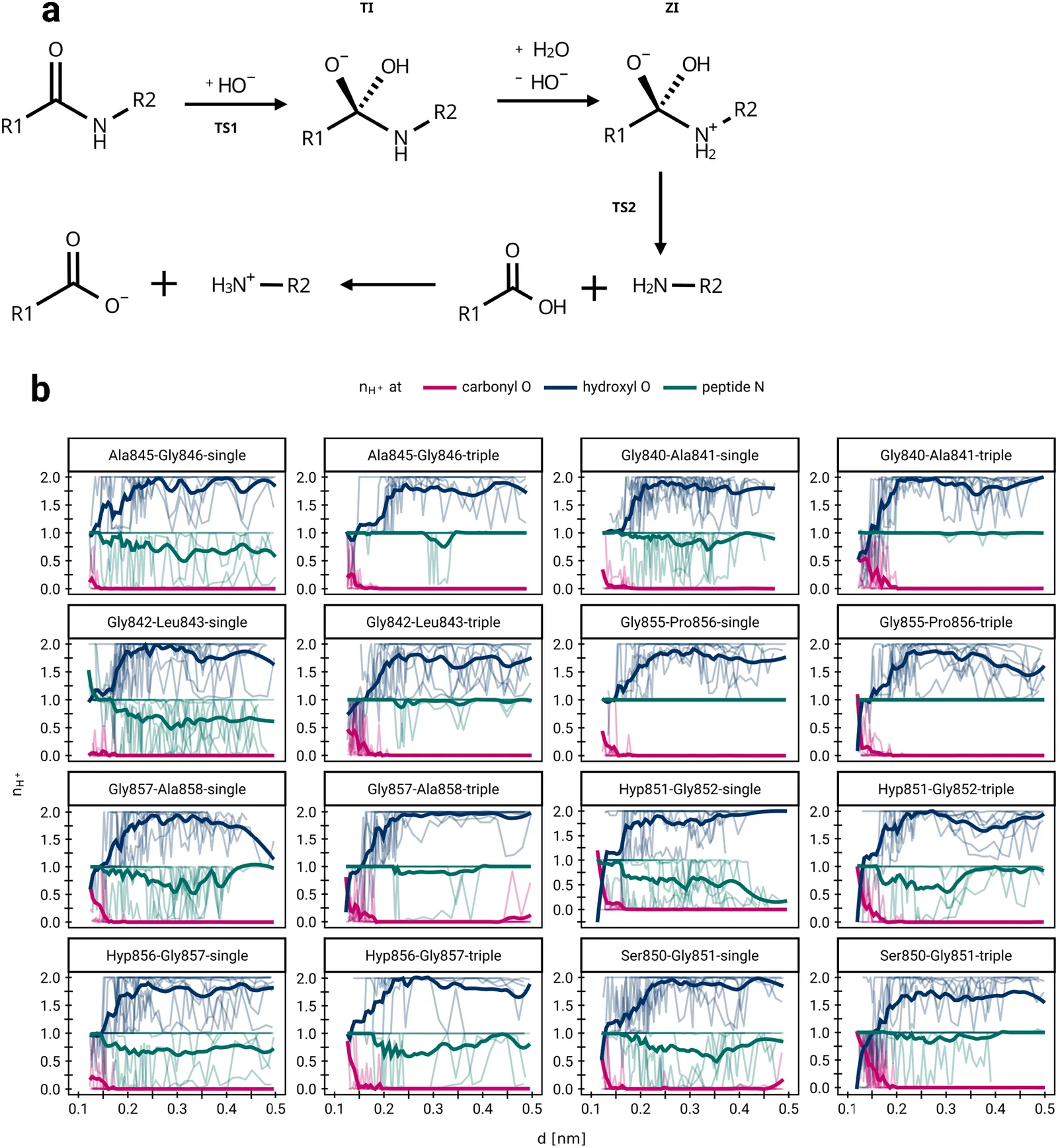
Assessment of spontaneous formation of a Zwitterionic Intermediate. (a) The complete reaction scheme of base-catalyzed hydrolysis with the inclusion of a Zwitterionic Intermediate (ZI) following.^21^ (b) Protonation states of the carbonyl O, the approaching hydroxide and the petide bond N during formation of the TI. Protons were uniquely assigned to the nearest proton acceptor. Data points represent averages within each window; thick lines indicate LOESS curves.

In the TI state, the original carbonyl oxygen and the nucleophilic oxygen become chemically indistinguishable; proton transfer events facilitated by the QM water allow either oxygen to be protonated. However, the umbrella sampling reached time scales likely not sufficient to equilibrate the protonation states, as only occasionally proton transfers are observed. Overall, the described proton dynamics are evident in both simulation systems, the isolated peptide and the triple helix, and there are no apparent differences between the two.

### The Triple Helix Impedes Hydroxide Attack

We next asked if, despite the alleged likelihood in the hydrolysis mechanism in the two systems, if the free energy profiles differ between the single and triple helix systems. Free energy profiles of the nucleophilic attack leading through the transition state (TS1) to the TI (Figure 1d) were computed relative to the educt state (defined at a reaction coordinate of 0.45(2) nm), using the Weighted Histogram Analysis Method (WHAM, see Methods). Profiles for the eight distinct hydrolysis sites along the peptide, for both the single fully solvated peptide and the triple helical system, are shown in Figure 6a. Based on the overall low bootstrapping errors, the profiles appear converged. We note, however, that protonation state changes are only observed partly (see section above), and are likely not sufficiently sampled in the TI. However, this does not affect the free energy profiles prior to the TI. The profiles feature a steady increase in free energy during nucleophilic attack and reach the transition state, a maximum in the free energy, at a C–O distance between 0.1 and 0.2 nm. The TI state is reached at a final C–O bond length of 0.1 nm. While all profiles plateau at the TI distance, not all exhibit a distinct minimum for the TI, probably due to an insufficient sampling of the proton transfers at the two oxygens of the TI.

**Figure 6:**
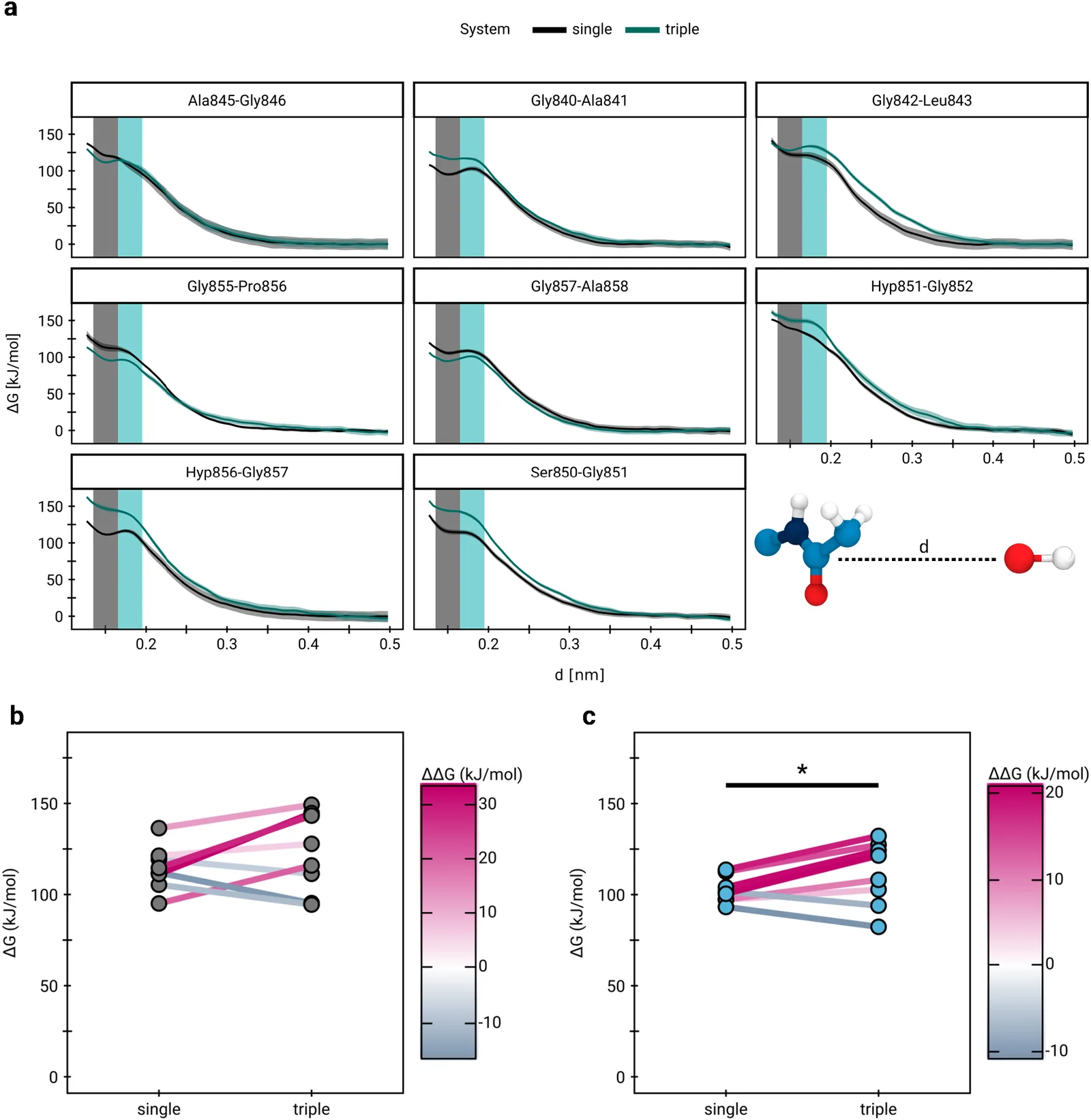
(a) Free energy profiles (WHAM) of the hydroxide attack (TS1). Cyan shading indicates the TS1 region; gray shading indicates the TI region. Energies are relative to the educt baseline. Shaded bounds represent the bootstrapping error. (b) Comparison of the reaction free energies of the TI for single peptide vs triple helix. (c) Comparison of the reaction free energy barriers of TS1. The star indicates statistical significance (α = 0.05). A paired *t*-test (*H*_1_: *Δ*Δ*G* > 0) yields *p* = 0.04.

The tetrahedral intermediate state leads to an oxyanion (Figure 1d, TI) that requires stabilization by hydrogen bonding with solvent molecules or protonation to the respective neutral form of the TI. We hypothesized that the presence of the triple helix destabilizes the TI, relative to the fully solvent exposed single peptide case, as the oxyanion might be less accessible to the solvent, and thus to hydrogen bond donors or proton donors. A pairwise comparison of the same site in the single peptide versus the triple helix context reveals no significant difference for the overall reaction free energy (Figure 6b). However, we observe a slight yet significant difference for the TS1 barrier (Figure 6c). The average activation free energy for TS1 was 102.5 ± 2.6 kJ/mol (±SEM) for the single chain and 111.6 ± 6.6 kJ/mol for the triple helix. The average difference (*Δ*Δ*G*) is 9.0 ± 4.4 kJ/mol. This difference corresponds to a decrease in the reaction rate by a factor of 30 to 40 according to the Arrhenius equation.^48^ Thus, embedding a peptide in a triple helix and thereby introducing two peptide chains as neighbors of the hydrolyzing peptide destabilizes slightly but measurably the transition state.

We next asked if the amino acid identities downstream and upstream of the hydrolysis site measurably effect the barrier for hydrolysis. When comparing the different profiles across the eight sites, no strong amino-acid-specific effects were observed (Figure 7a–d). However, interestingly, we find Pro-X and Hyp-X peptide bonds to be slightly kinetically stabilized (Figure 7d), suggesting that prolines N-terminal of the peptide bond, with their strong tendency to remain in a polyproline II configuration, might partly hinder hydrolysis.

**Figure 7:**
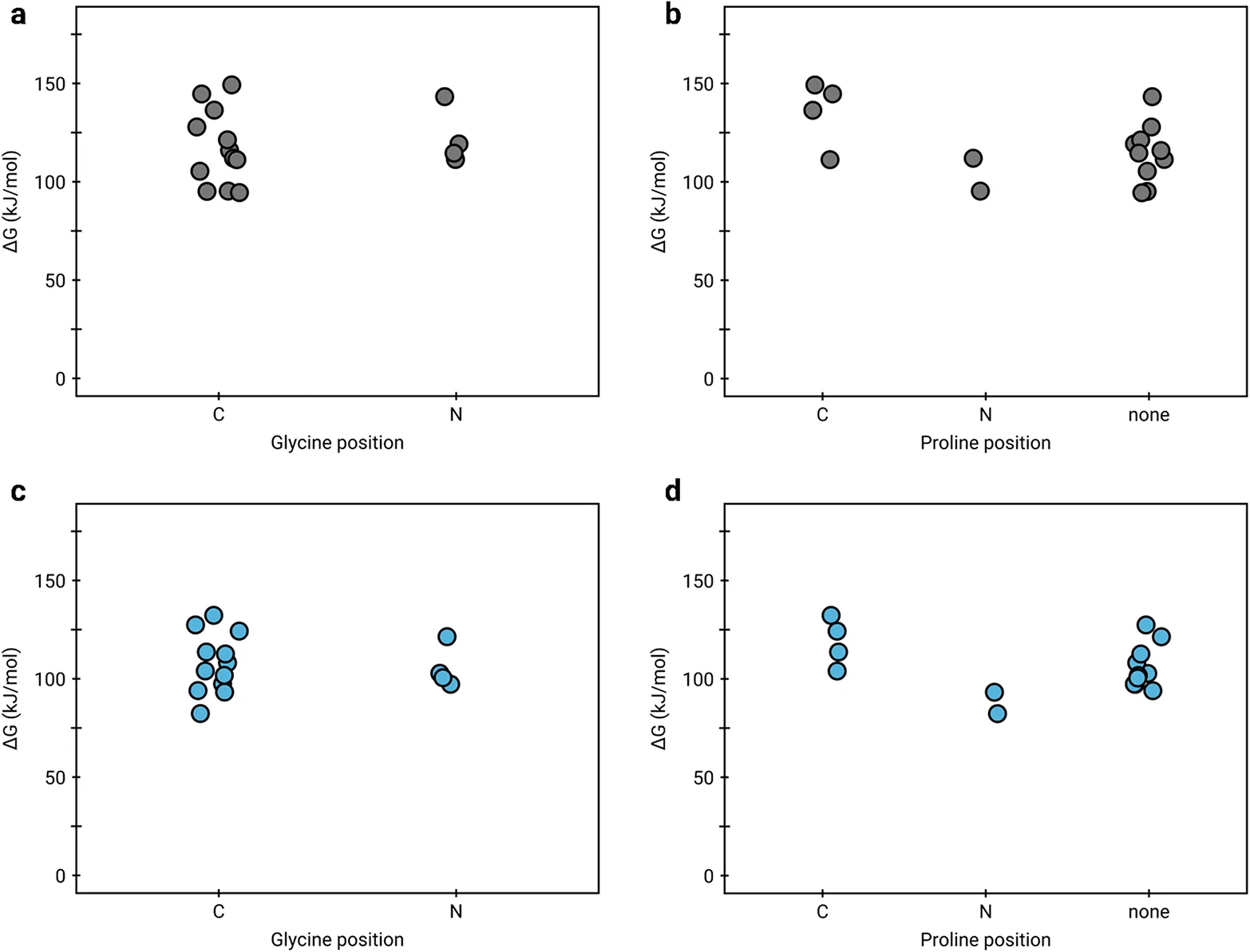
Influence of adjacent amino acids on reaction free energies. (a) Comparison of the reaction free energies of the TI conditioned on the position of Glyine in the peptide bond. (b) Comparison of the reaction free energies of the TI conditioned on the position of Proline in the peptide bond. (c) Comparison of the reaction free energies of TS1 conditioned on the position of Glycine in the peptide bond. (d) Comparison of the reaction free energies of TS1 conditioned on the position of Proline in the peptide bond.

## Discussion

Our hybrid QM/MM simulations provide atomistic insights into base-catalyzed hydrolysis within the collagen triple helix. The results, based on extensive sampling, highlight the critical role of solvent dynamics and proton mobility. The unconstrained nature of the chosen approach is a double-edged sword. It enabled sampling of interesting regions of phase space and direct observation of physically relevant behavior, such as proton mobility in the QM region during the reaction. However, the broad sampling makes it harder to attribute the results to any specific structural effect without even higher computational costs. Nonetheless, the plethora of QM/MM trajectories with a total sampling time of 22.7 ns of the entire hydrolysis reaction presented here provide a great starting point that can be used e.g., as training data for machine learning approaches to more efficiently emulate hydrolysis in complex systems^49^ and as a reference system.

Previous single molecule force spectroscopy experiments and quantum chemical calculations have revealed unprecedented details of how force accelerates peptide bond hydrolysis.^21^ Direct quantitative comparisons of the energetics are elusive, as our MD simulations yield free energies and thus include entropic contributions, while the earlier study is based on electronic energies of minimized structures. Still, both approaches support the conclusion that at a force of 1 nN, the formation of the tetrahedral intermediate represents the rate limiting step. Our umbrella sampling suggests, for the single peptide case, on average around 100 kJ/mol for the TS1 barrier and 20–60 kJ/mol (with a high uncertainty due to limited sampling) for the TS2 barrier, compared to ∼80 and ∼65 kJ/mol from the previous static QM calculations, respectively.

We have previously observed homolytic cleavage in the triple helices of collagen.^13,22^ Homolysis and hydrolysis can kinetically compete with each other as the two potential reaction channels leading to peptide chain rupture under force. Given that collagen is very hydrated and water surrounds each triple helix (Figure 8), together with the fact that force can strongly accelerate peptide bond hydrolysis, the observation of homolysis versus hydrolysis is surprising. We show here that the triple helix structure can slow down hydrolysis to some extent, thereby potentially allowing homolysis to become competitive.^49^ Comparison of free energy profiles between single peptides and the triple helix reveals that the helical structure creates an additional kinetic barrier to hydrolysis. Specifically, the 9 kJ/mol increase in the activation energy for the triple helix reduces the reaction rate by a factor of 30–40. A likely explanation for these results is the *n* → π* interaction between the peptide bond, which has previously been shown in model compounds to kinetically impede hydrolysis by 1–2 orders of magnitude.^23^ This interaction is particularly strong in the triple helix’ polyproline II configuration and supported by the prevalence of prolines and hydroxyprolines in collagen, but diminished in an extended single peptide under force. Homolysis, instead, is enhanced by the fibrillar structure of collagen, as stress concentrations accelerate this reaction more strongly than hydrolysis. Taken together, the QM/MM results presented here add to our understanding of collagen and more generally protein mechanochemistry, and provide a step toward simulating the direct competition between alternative reaction channels in stretched protein materials.

**Figure 8:**
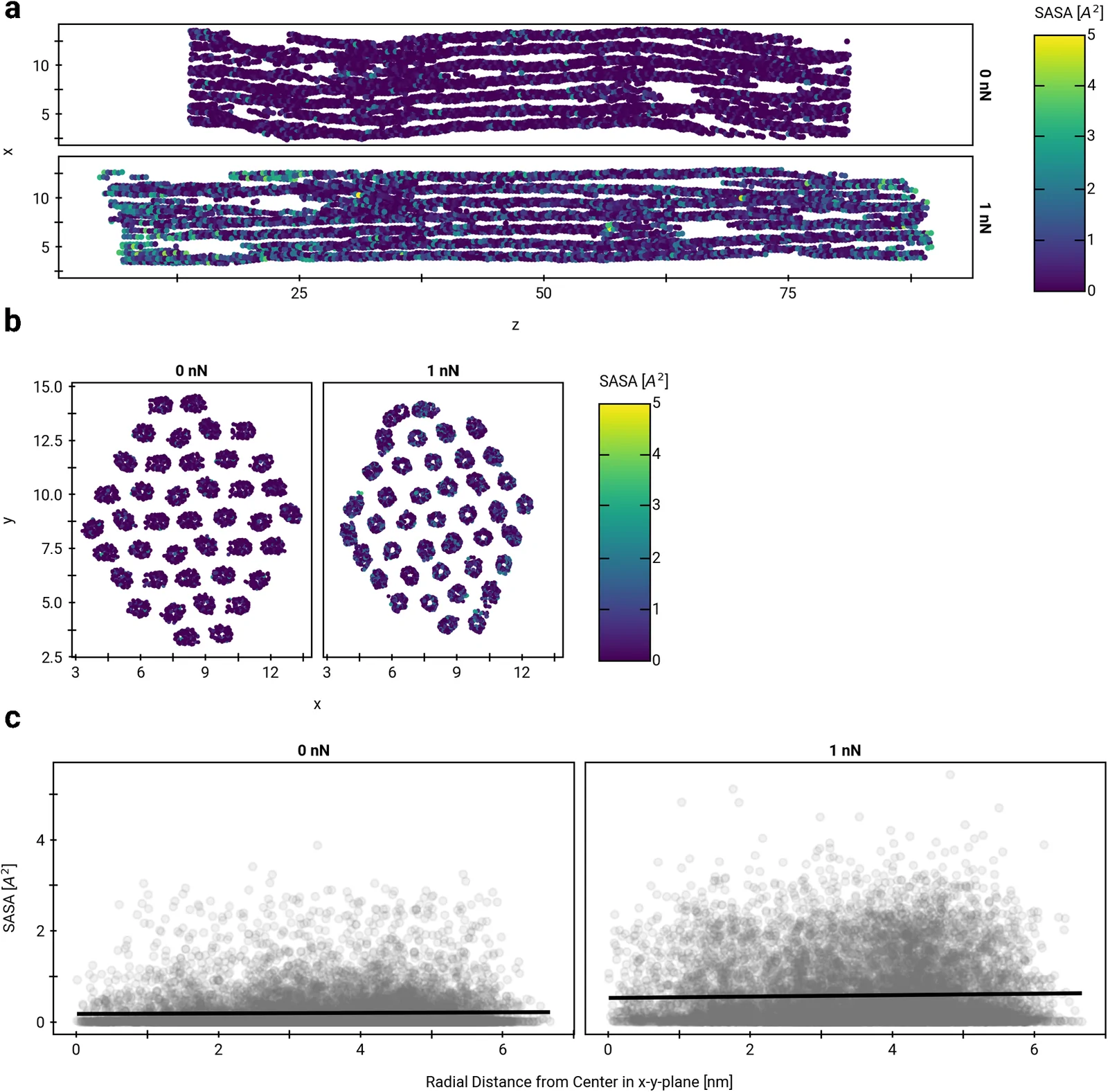
A collagen fibril simulated under 1 nN external force. (a) A slice through the collagen fibril in the *x*–*z*-plane with y-coordinates filtered to be between 7.9 and 9.6 nm. Each peptide bond is colored by Solvent Accessible Surface Area (SASA) for the 0 nN and 1 nN shear pulling setup. (b) A slice through the collagen fibril in the *x*–*y*-plane with z-coordinates filtered to be between 40 and 50 nm. Each peptide bond is colored by Solvent Accessible Surface Area (SASA) for the 0 nN and 1 nN shear pulling setup. (c) SASA of the peptide bonds in the overlap region of the collagen fibril (z-coordinate between 30 and 60 nm) plotted against their radial distance from the center in the *x*–*y* plane. Black lines are linear models. A peptide bond near the center of the fibril is equally hydrated as one near the surface, especially under force.

### Conclusion

In summary, our QM/MM free energy calculations show that embedding a peptide within the collagen triple helix raises the rate-limiting barrier for base-catalyzed hydrolysis by 9 kJ/mol, slowing the reaction 30- to 40-fold relative to an isolated chain. This kinetic protection, attributable to the *n* → π* interaction characteristic of the proline-rich polyproline II conformation, helps explain why force-driven collagen failure can proceed through homolytic radical formation rather than hydrolysis. More broadly, the approach demonstrates how secondary structure modulates peptide-bond chemistry, offering a route toward simulating competing reaction channels in mechanically stressed protein materials.

## Data Availability

The complete simulation and analysis code is available at github.com/graeter-group/hydrolysis-qmmm-workflow. The complete simulation and analysis code for the supplementary SASA analysis is available at github.com/graeter-group/kimmdy-hydrolysis-examples. Raw simulation data are available from the corresponding author upon reasonable request.
